# “*It gives you a road map of what to do to solve your problems”:* acceptability of a combination HIV prevention intervention among adolescent girls in Uganda

**DOI:** 10.1186/s12889-023-15083-2

**Published:** 2023-02-06

**Authors:** Ozge Sensoy Bahar, Proscovia Nabunya, Flavia Namuwonge, Satabdi Samtani, Vicent Ssentumbwe, Florence Namuli, Natasja Magorokosho, Fred M. Ssewamala

**Affiliations:** 1 Brown School, Washington University in St. Louis, St. Louis, MO USA; 2 International Center for Child Health and Development, Brown School, Washington University in St. Louis, St. Louis, MO USA; 3 International Center for Child Health and Development Field Office, Masaka, Uganda

**Keywords:** Adolescent girls, HIV prevention, Combination intervention, Intervention acceptability, Qualitative, Sub-Saharan Africa

## Abstract

**Background:**

The HIV burden remains a critical public health concern and adolescent girls are at significantly higher risk compared to the general adult population. Similar to other sub-Saharan African countries, Uganda reports high HIV prevalence among adolescent girls and young women. Yet, both evidence-based HIV prevention interventions and their acceptability among adolescent girls have not been widely studied. In this study, we examined the acceptability of the Suubi4Her intervention, an evidence-based combination intervention aimed at reducing HIV risk among adolescent girls in Uganda.

**Methods:**

We conducted semi-structured in-depth interviews with 25 adolescent girls upon intervention completion to explore their experiences with the Suubi4Her intervention that was tested in a clinical trial in Uganda (2017–2023). Specifically, we explored their decision-making process for participating in the intervention, experiences with program attendance, and their feedback on specific intervention characteristics. Informed by the Theoretical Framework of Acceptability, the data were analyzed using thematic analysis.

**Results:**

The main motivation for participation was access to health-related information, including information on STIs, HIV, and pregnancy as well as information on banking, saving, and income-generating activities. Though many participants did not have any initial concerns, mistrust of programs, initial paperwork, caregiver’s ability to commit time, concerns about ability to save, and HIV/STI and pregnancy testing were raised by some participants. Facilitators to session attendance included motivation to learn information, caregiver commitment, reminder calls, and incentives received for participation. The main challenges included household responsibilities and obligations, difficulty raising transport money, and weather challenges. Adolescent girls appreciated the group format and found the location and times of the sessions convenient. They also found the content relevant to their needs and noted positive changes in their families.

**Conclusions:**

The results showed high intervention acceptability among adolescent girls. These findings have important programmatic and policy implications in Uganda, especially given the higher HIV prevalence among adolescent girls in the country.

**Trial registration:**

NCT03307226 (Registered: 10/11/17).

## Background

Sub-Saharan Africa (SSA) continues to be heavily impacted by the HIV epidemic, with 67% of the people living with HIV residing in the region [1]. In addition, six in seven new HIV infections among adolescents (aged 15 to 19 years); and girls and young women (aged 15 to 24 years) are twice as likely to be living with HIV than men in SSA [1]. Dropping out of school increases young girls’ vulnerability to HIV as it is associated with several risk factors, such as age-disparate and transactional sex, early marriage, inconsistent condom use, and limited ability to negotiate safe sex [2–7]. In addition, mental health challenges, such as depression, low self-esteem and anxiety are shown to be associated with age-disparate and transactional sex among young girls [8, 9]. Moreover, higher depression among young females has been associated with co-factors of HIV risk [10]. Hence, it is critical to address these complex and multilayered risk factors among this vulnerable population given their heightened risk for HIV infection.

In many SSA countries, including Uganda, household financial stability is strongly associated with access to education, a protective factor [11], especially for adolescent girls [12–15], where families may prioritize male education when resources are limited [16–18]. Household financial stability also influences the quality of family relationships where poverty negatively impacts parent–child communication and involvement [19–21], another protective factor for adolescent sexual risk taking and mental health [22–27]. Relatedly, providing families with opportunities to strengthen their economic stability and family supportive processes through combination interventions that incorporate economic empowerment components may be critical in minimizing sexual risk-taking behaviors, reducing school dropout, and addressing mental health stressors among adolescents.

While there are studies testing the effectiveness of interventions targeting youth mental health or HIV prevention in low and middle-income countries (LMICs), intervention acceptability studies among adolescents in LMICs, and specifically in Africa, are still limited [28]. This is particularly critical as interventions have higher uptake and effectiveness if adolescents find them acceptable [29, 30]. A recent systematic review of peer-reviewed studies assessing intervention acceptability with youth (aged 10–24) in Africa mostly included interventions focused on HIV- related or sexual and reproductive health-related outcomes [28]. Despite the diversity of intervention setting, type, and delivery mode, common themes that emerged as reasons for acceptability included the product or intervention being easy to use, intervention knowledge or knowledge provided by the intervention, greater autonomy as a result of the intervention, feeling supported during the intervention, and reassurance that their privacy and confidential information would be protected [28]. On the other hand, reasons for ‘unacceptability’ included conservative views about the intervention or its content; concerns around intervention cost, access, and inclusiveness; fear of pain and side effects (for biomedical interventions); stigma or distrust; and lack of knowledge or support [28].

While this review provides important insights into factors that increase intervention acceptability, all interventions were either single interventions or only explored the acceptability of one component of a combination intervention, leaving out the opportunity to assess the acceptability of an intervention as a package. Other acceptability studies, in the context of HIV prevention in particular, primarily focus on pre-exposure prophylaxis (PrEP) uptake in the form of facilitators and barriers [31–33]. Hence, more studies examining the acceptability of combination interventions, including those that incorporate economic empowerment and target multiple risk factors in HIV prevention are warranted.

Against this backdrop, in this study, we explore the acceptability of a combination intervention named Suubi4Her (Suubi means hope in Luganda, the local language in the study area where the intervention was delivered) that tested the impact of Suubi4Her on reducing HIV risk among adolescent girls in Uganda.

## Methods

### Overview of the randomized clinical trial

The Suubi4Her study, a five-year National Institute of Mental Health (NIMH) funded study, is a 3-arm cluster randomized clinical trial (2017–2023) that evaluates the impact of a combination intervention on reducing HIV risk among high school adolescent girls in Uganda. The intervention, titled *Suubi4Her,* combined savings-led economic empowerment through youth development accounts (YDA) and evidence-based family strengthening intervention delivered via multiple family groups (MFG). The three study conditions are: 1) Control condition: bolstered usual care that involves standard health and sex education, provision of textbooks, notebooks, and pens; 2) Treatment condition 1: YDA, Financial Literacy Training (FLT) and Income-generating Activities (IGA); and 3) Treatment condition 2: YDA-FLT-IGA + MFG. The study enrolled 1260 school-going adolescent girls (14 to 17 years of age) across 47 secondary schools located in five geopolitical districts, Rakai, Kyotera, Masaka, Lwengo, and Kalungu within the greater Masaka region.

Adolescents were eligible to participate if they met the following inclusion criteria: 1) female; 2) aged 14–17 years; 3) enrolled in the first or second year of secondary school; and 4) living within a family (broadly defined) and not an institution or orphanage, as those in institutions have different familial needs (please see references [34, 35] for more details about the recruitment process for the clinical trial).

As part of the randomized clinical trial, a qualitative component was included to explore: a) participants’ experiences with the intervention in each treatment condition (e.g., challenges and benefits participants experienced), including individual, family, and contextual factors that may have hindered and/or facilitated youth’s experiences in each treatment arm (YDA-FLT-IGA + MFG, YDA-FLT-IGA only) and perceptions on program sustainability; and b) key multi-level factors (individual, family, programmatic, cultural) that may have impacted participants’ observable behaviors and decision-making in regard to savings, mental health, and sexual risk taking. In this study, we examine adolescent girls’ experiences with the Suubi4Her combination intervention (YDA-FLT-IGA + MFG).

### Study setting

Uganda is one of the SSA countries hardest hit with HIV with unprecedented numbers of ALHIV (over 170,000) [36]. According to Uganda Ministry of Health, the prevalence of HIV among females ages 15 to 49 in Uganda was almost twice as high (6.8%) as the HIV prevalence among males (3.9%) in the same age group in 2020 [36]. The study was conducted in the Greater Masaka region of Uganda, with an 11.7% prevalence (as opposed to 5.4% nationally) for adults ages 15 to 49 years old [36].

### Description of the combination intervention components

#### YDA component

In addition to the sexual health education (per the National Sexuality Education Framework)provided in all secondary schools in Uganda, adolescent girls in this study arm received a family economic empowerment intervention that included incentivized YDA held in the adolescent's name together with their caregiver in a financial institution. The savings in the YDA were matched by the program with a ratio of 1:1. They were allowed to invest up to 30% of their total matched savings in a family-based income-generating business. The remaining 70% of the savings were to be used to support the education and skill development of adolescent girls. Participants’ access to the matching funds was contingent upon them completing the 4-session manualized FLT sessions offered together with YDA [37].

FLT sessions were delivered by trained facilitators. Each session (see Table 1) was conducted at the participants’ schools when classes were not in session, including on weekends. The sessions lasted approximately one hour and were delivered in Luganda (the local language used in the study region).

**Table 1:** Financial literacy training

Session #	Content
Session 1	overview of financial literacy and budgeting
Session 2	saving, asset building, and asset accumulation
Session 3	bank services in the community
Session 4	debt management, borrowing money, cost of borrowing, and the dangers of defaulting

In addition to financial literacy, participants were provided with goal-specific IGA training. This was specifically on crop husbandry (e.g., backyard gardening in fruits and vegetables), animal husbandry (e.g., rearing pigs, chickens and rabbits), tailoring, weaving baskets, and knitting. Participants also received information on how to market their products resulting from IGAs and manage a small kiosk. Finally, participants were linked to a local marketing agency that bought their finished products.

### MFG family strengthening component

The MFG family strengthening intervention, called Amaka Amasanyufu (Happy Families in Luganda, local language in the study area), is an evidence based 16-session manualized intervention delivered by trained parent peers and community health workers. The intervention is organized around six constructs: four Rs (Rules, Responsibility, Relationships, and Respectful Communication) and two Ss (Stress and Social Support) that target parenting skills and family processes. The intervention aims at building support for families through opportunities for caregivers and children to communicate in a safe setting with other families who have similar experiences, thus allowing families to benefit from each other’s contributions [38]. The manual was culturally adapted from the U.S. version called “4Rs and 2Ss” intervention for another study in Uganda [39]. For the Suubi4Her study, session 3 was revised to include a session focused on puberty and knowledge on HIV and sexually transmitted infections (STIs).

The multiple family groups involved 15 to 20 families, with the adolescent participant and at least one adult caregiver to the target participant present in each session (e.g., caregivers, siblings, or other extended family members). Lasting approximately one hour, sessions included group discussions, role-plays, and other activities that foster support, learning, and interaction both within and among the families participating in the sessions. The sessions were delivered bi-monthly in schools on days when classes were not in session (e.g., weekends, school holidays). Sessions were facilitated by two facilitators, specifically community health workers and peer parents already trained in MFG intervention as part of the NIMH-funded SMART Africa study [39].

### Qualitative sampling for the study

A stratified purposeful sampling was utilized to select the qualitative sample using three key outcomes (sexual risk taking, mental health, and matched savings) in the combination intervention condition (*n* = 381 adolescent girls). Specifically, we used biomarker (STI and HIV) data to stratify participants who screened positive (new cases) and negative at 12-month follow-up for sexual risk-taking.. We stratified participants in the lowest and highest quartiles in mental health using theBeck’s Depression Inventory scale [40] and savings using YDAs tracked through monthly bank statements. We then randomly selected participants from each of the stratified groups. A total of 27 adolescent participants participated in the interviews from the combination intervention arm (YDA-FLT-IGA + MFG). We excluded two participants from the analysis for this study as they did not participate in any of the intervention sessions and would not be in a position to answer intervention-related questions. Both participants stated that they were pregnant and their homes were far from where the sessions were delivered. Pseudonyms are used to protect participant confidentiality.

### Data collection

Semi-structured in-depth interviews were conducted following intervention completion between September 2020 and January 2021 in a private setting at participants' school. The interview guide explored: 1) participants’ experiences with the respective intervention and its specific components (i.e., YDA, FLT, IGA and MFG); 2) multi-level factors that may have impacted participants’ observable behaviors and decision-making on savings, mental health, and sexual risk taking. Interview questions used for this study included, but were not limited to: *“Could you tell us a little bit about why you decided to participate in the program?”, “What parts of the project, if any, made you hesitate to take part?”, “What information or skills covered have been most helpful to you?”, “What were the things/factors that made it easier for you to attend the sessions?” “What were the factors that made it difficult for you to attend the sessions?”* and “W*hat did you think of the way the sessions were delivered?”* Probing questions were used where relevant.

All interviews were conducted face to face in Luganda, the local language in the study region. The interview guide was translated from English to Luganda and back-translated by team members fluent in both languages. Interview questions were then reviewed and revised –where necessary- by the study team that comprised research assistants and co-investigators fluent in both languages and pre-tested. This process ensured that interview questions sounded natural and conversational, and conveyed the meanings intended. Interviews were conducted by research assistants fluent in both languages and trained by two authors with qualitative research expertise. Interviews lasted approximately 2 h on average (ranging from 1 h to 2:48 h) and were audiotaped. Field notes were taken upon completion of the interview.

### Theoretical framework guiding the data analysis

We used Sekhon et al.’s [29] theoretical framework of acceptability (TFA) to guide our analysis. TFA defines acceptability as a multi-faceted construct that seeks to understand to what extent people receiving a healthcare intervention consider it to be appropriate, based on cognitive and emotional reactions to the intervention [29]. More specifically, TFA consists of seven constructs, namely affective attitude, burden, perceived effectiveness, ethicality, intervention coherence, opportunity costs, and self-efficacy [29]. Affective attitude refers to how an individual feels about the intervention, prior to (anticipated) and after participating (experienced) in the intervention. Burden includes the perceived amount of effort that would be required to participate in the intervention (anticipated) and the actual amount of effort that was required to participate (experienced). Perceived effectiveness also includes both the extent to which the intervention is perceived to be likely to achieve (anticipated) and to have achieved (experienced) its purpose. Ethicality is the extent to which the intervention aligns with the value system of the participating individual. Intervention coherence refers to the extent to which the participant understands the intervention. Self-efficacy is the participant’s self-confidence that they can perform the behaviors that are required for intervention participation. Finally, opportunity cost also includes both the extent to which benefits, profits, or values one must give up engaging in the intervention (anticipated); and the extent to which one gave up when engaged (experienced).

### Qualitative data analysis

Interviews were first transcribed verbatim and then translated from Luganda to English by research assistants fluent in both languages. Dedoose analytic software was used for data analysis. We used inductive techniques for thematic analysis of the data [41–43]. Specifically, three interview transcripts were randomly selected, read multiple times, and independently coded by two team members as the first step for a codebook. Both sensitizing concepts informed by the existing literature and the content of the intervention as well as identifying emergent themes (open coding) were used [44]. Broader themes were broken down into smaller, more specific units until no further subcategory was necessary. Initial codes were discussed with the first author during team meetings and reorganized when necessary to create a final codebook that was used to code all transcripts. Once coding inter-reliability was accomplished, interviews were coded independently by two team members.

The analysis, conducted by two authors, compared and contrasted themes and categories to identify similarities, differences, and relationships among findings. Peer debriefing and audit trail were used to ensure rigor [39, 40]. The codes and the findings were presented to two members of the research team who were not involved in the data analysis to discuss the plausibility of themes and related findings [39, 40].

### Ethical considerations

Participation in the study was voluntary. Written consent was obtained from caregivers for the adolescent girls to participate in the study. Written assent to participate was obtained from the adolescent girls upon receiving their caregivers’ consent. The consenting and assent activities were conducted separately for the adolescents and caregivers to avoid potential coercion. Study procedures were approved by the Institutional Review Board at Washington University in St. Louis (IRB #201,703,102), the Uganda Virus Research Institute (GC/127/17/07/619), and the Uganda National Council of Science and Technology (SS4406). The parent randomized clinical trial is registered in the clinical trials database (NCT03307226).

## Results

### Participant characteristics

Fifty-two percent (52%) of the participants were 16 to 17 years old. Twenty percent (20%) of the adolescent girls were orphaned. The primary caregiver for 64% of the sample was the biological parent. The number of people living in the household ranged between 2 and 13 (see Table 2).

**Table 2:** Participant demographics

Variable	Total Sample (*N* = 25) % (*n*)
***Age***	
14 to 15 years	48.00 (12)
16 to 17 years	52.00 (13)
***Orphanhood Status***	
Orphan	20.00 (5)
Non—orphan	80.00 (20)
***Primary Caregiver***	
Biological parent	64.00 (16)
Grandparent	8.00 (2)
Other relative	28.00 (7)
***Household size (Mean, SD)***	
Number of people in HH (min/max = 2–13)	6.48 (3.24)

### Intervention acceptability

To capture the full picture in terms of acceptability of the combination intervention for the participating adolescent girls, we explored their decision-making process, specifically their motivations to and concerns about participating in the program prior to enrolment as well as the barriers and facilitators to their participation after enrolment. We also inquired into their thoughts regarding the different characteristics of the intervention, including group format, day and time of the sessions, and session facilitators (see Table 3. Themes and subthemes).

**Table 3:** Themes and subthemes

Themes	Subthemes
Motivation to participate in the program	•Access to health-related information* (HIV, STIs, pregnancy)* •STI testing •Opening bank accounts •Matched savings •Income-generating activities •Potential for better family relations
Initial concerns	•Putting together the necessary documents (i.e. birth certificate) •Mistrust in programs •Time commitment •Potential inability to save •Testing (STI and pregnancy)
Facilitators to attendance	•Desire to learn more information •Reminder calls •Caregiver commitment •Incentives •Transport refund •Lunch
Challenges	•Other responsibilities and obligations (e.g., family, school work) •Difficulty raising transport money •Long distances •Weather challenges •Lack of interest
Intervention characteristics	
Group format	•Shared experience •Knowledge sharing and learning •Gaining confidence in public speaking •Concern about confidentiality
Session attendance with caregivers	•Co-learning •Reconnecting with caregivers •Changing caregiver perceptions •Separate activities for adolescents and caregivers
Delivery location	•Convenience •Quietness •Spaciousness •Lack of bathroom nearby
Day and time of delivery	•No conflict with other obligations •Interference with other obligations •Inconvenient early start time
Facilitators delivering the sessions	•Inclusive •Knowledgeable and well-trained •Respectful •Patient •Having two facilitators •Good relationship among facilitators
Content relevance	•Information on income-generating activities •Information on budgeting and saving •Information on STIs and HIV •Information on family relations and communication •Applying the skills and knowledge learned

### Decision to participate in the Suubi4Her program

#### Motivation to participate in the Suubi4Her program

There were multiple reasons that attracted adolescent girls to participate in the program. For many, it was not just one aspect but a combination of aspects that motivated them to enroll. Information related to HIV, STIs, and pregnancy, including the testing offered as part of the program, was a reason for many participants. For instance, Gloria wanted to learn about *“HIV, how a person gets infected with HIV, how to prevent it, and how a person can avoid unwanted pregnancies”.* Annet also talked about STI testing as one of the most appealing aspects of the program in addition to learning “*certain things that my caregiver may be afraid to tell me*”. With the testing, she could know her status and take the necessary action, whether that would be *“keep adhering to my medication so I can recover soon and when I am healthy, I can maintain it by protecting myself.”* She also added that the prospect of being able to pay her school fees by saving and receiving the matched saving was enticing to her and elaborated on the struggle of paying school fees.

*Annet: It [school fees] is hard for us. They send you back from school and sit for a whole week; by the time you go back to school, your friends have already covered things and it’s hard for you to understand the content.*

The economic empowerment component was of interest to many participants. For instance, in addition to learning about STIs and pregnancy, Margret was also motivated by the fact that the program would help participants with opening bank accounts. She thought that this would be an invaluable opportunity for her to start saving and using for her needs, including education.

*Margret: They told us that, they are going to open for us accounts in the bank because, for me at first, I did not know that even a person (laughs) who does small jobs can also have an account in the bank. And this itself attracted me to register and join. Because to open up the account in the bank you feel like, it’s very precious…..When you earn five thousand or three thousand [Uganda shillings], you can send [deposit] it and keep it in your account and then use it in the future, to help you in your education, to buy necessities and others.*

In addition to being excited to learn knowledge that *“our parents cannot teach us”,* Annah was interested in the matched savings and income generating activities to be offered. She pointed out that in addition to the matched savings being helpful for school fees, opportunities to learn about the importance of saving and how to start small business informed her decision to join.

*Annah: The other thing is you get to know that not every money you get, you to have to eat [spend it] it. That is not a good thing. You can start a small business and when you get money after selling something from the business you can buy yourself something like clothes and topping up on school fees together with your parents.*

Though discussed less frequently, some participants identified the focus on family relations as a motivation. For instance, Sarah mentioned sharing about her family relations in addition knowing her HIV status and *“learning new things concerning the girl child”.* Similarly, Flora expected that the program would improve her relationship with her parents. Joseline also was motivated by the opportunity to learn about *“a happy family, the way we can relate to friends and neighbors”.*

### Initial concerns about participating in the Suubi4Her program

Adolescent girls talked about what made them hesitate to participate when they first heard about the Suubi4Her program. More than half of them mentioned that they had no concerns. However, while one of the participants did not have any concern about participation, she noted the challenges of putting together the documents needed for account opening such as birth certificates and passport size photos. She worried that this might prevent her from joining the program but was able to get all the documents with help from her father.

*Racheal: I wasn’t sure I would get them [the required documents] or not. I prayed to God to enable me to enroll into the organization…. My dad took me to a distant place to obtain passport size photos and I also got the birth certificate.*

This was echoed by Margret who lived with her grandmother and her birth certificate was with her mother who lived far away. However, she had an immunization card with her birth date that she was able to use instead. She also added that she was hesitant about joining initially because she heard of programs that did things differently than what they originally presented.

*Margret: I heard that there are certain organizations that come to school and bring something like this with an aim which is very different. Just like they talk about this and this in the office yet their aims are very different and after registering students they do different things.*

Anisha shared a similar concern where her parents thought that the Suubi4Her program might be a *“cheat”* that would not deliver on what it promised and instilled doubt in her mind.

*Anisha: Because my parents said “we might take that child into that program where they are claiming they will open for her an account and yet they are cheats.” Since older people have had more life experiences, you also start to think these people might be cheats considering they are the ones who give us our bank account numbers, and if you want to withdraw you have to reach them first. This made me hesitate a bit.*

However, she was convinced by her older sister’s determination to participate as well as the program staff’s reassurance that her savings would not be stolen, but rather matched. Violet also mentioned that some of the students at her school thought *“this was a fake program”.* Two participants, Ruth and Mariam, had initial concerns about their caregivers. Ruth was worried that her parents would not have the time to attend the program sessions because of their busy schedule. This was critical because the program was a family-level intervention that required caregiver attendance together with their child. She later was able to convince her caregiver to attend the sessions.

*Ruth: What made me hesitant was when they told us to invite our guardians. Many parents do not have time to go to school, especially on project-related issues, which they don’t even know about. It was a challenging situation for us because some of our parents spend time at work and they think coming to school is just disrupting them.*

Two participants had concerns about whether they would be able to deposit or save during the program. Harriet shared that her father had another child in college that he was paying fees and expenses for. Hence, she was worried that there might not be enough money left for her to save in her account.

*Harriet: What made me hesitate was that part of saving. Okay, money wouldn’t be a problem but my father has another child who is a university student who was taking millions, so telling him about that saving stuff was going to be difficult.*

Finally, two participants were concerned about the testing for STIs, HIV, and pregnancy, though for different reasons. For Gloria, the concern was about potentially finding out about a positive pregnancy test and the shame associated with it.

*Gloria: As girls, you may be afraid of your health status because you might have done something like having sex unintentionally, and in case you’re informed that you’re pregnant, you feel ashamed. That might have made me afraid.*

Annet, on the other hand, was afraid of “*being pricked all the time*”. When she consulted with a friend, her friend reassured her that the blood sample will not be withdrawn all the time and that the needle would be small, after which she said “*let's go and enroll so we can know our status.*

### Program attendance

#### Facilitators to attendance

One of the main facilitators that enabled adolescent girls’ attendance to both FLT & IGA and MFG sessions was the desire to learn and gain new knowledge. When talking about FLT and IGA sessions, Harriet stated that she was motivated to attend the sessions because she was interested in learning about savings and ways to manage their expenditures, “*What made it possible for me to come for the sessions was to learn how to save, spend money where necessary, for example buying food instead of a dress of my choice.”* Annet noted that she wanted to learn about what she could do to generate profits with the little money she had.

*Annet: What enabled me to attend the sessions was because I was interested in what I am doing. I wanted to give in some time to learn and to know the things I can start up, with a little amount of money and generate huge profits.*

After attending the first session where she learned about saving and budgeting, Ruth indicated that it motivated her to continue attending the rest of the sessions.

*Ruth: What made it easy for me to attend the sessions is that after I attended the first session, I enjoyed it because they were training us about saving and the bank stuff, including budgeting for your money. After that, I decided to start attending the training sessions and made sure I get everything they were telling us.*

Participants also mentioned the motivation to learn new things as a facilitator to attending MFG sessions. For example, Ruth was motivated to learn how to better communicate with her guardian, which motivated her to attend the MFG sessions: *“The other things that enabled me to attend these training sessions were to learn how to communicate with my guardian, how I can approach him, and indeed, these sessions improved our morals.”* Margret stated that despite the temptation of staying home and sleeping on a Saturday morning, she chose to come to sessions because “*there is a lot to learn from there.”*

Another facilitator to session attendance was being reminded ahead of time via phone calls by the team. Harriet noted that they received calls from the team for both FLT&IGA and MFG sessions:

*Harriet: They used to first communicate that on this particular day we have financial literacy training and other sessions, let it be respectful communication sessions, and I could come well prepared to listen and learn from the sessions.*

Similarly, Janet talked about doing her chores the day before when she received the reminder call to make sure she made it on time to the sessions. She added that her father made sure to inform her when he was informed.

*Janet: They used to inform us earlier on and this enabled us to do whatever we had to do a day before, such that we reduce the workload for that day of the sessions and to reach on time…We used not to be occupied at home and whenever dad was informed, he used to inform us as well.*

Other participants also talked about their parents’ commitment. For instance, Ruth’s caregiver encouraged her to attend the sessions: "*He could remind me and would ask me to attend every time because they were training about interesting things.”* Anisha talked about her caregiver’s readiness to attend the sessions, whenever they were contacted*, “Whenever my caregiver would be called and asked to come with me, she would leave whatever she was doing and come to attend the sessions.”*

Participants also acknowledged the incentives provided to them as a major facilitator to continue attending the sessions. Specifically, participants were provided with lunch, and they were given a transport refund for every session they attended. Many participants appreciated the transport refund as this made it easy for them to continue attending the sessions. For example, Sarah noted that it was easy to attend because lunch and transportation to the venue were provided. Indeed, Annah noted that the money motivated her to attend the session, as she would later use that money to buy chickens to generate income in the future.

*Annah: What enabled me to attend them, first of all, they would give us some money which I could use to buy chicks and rear at home. After some time, when God wills, they would lay eggs and produce other chicks which would also eventually grow. So even if you bought like two chicks, they would multiply then you sell and get some money…*

She went on to add that a “well-balanced diet lunch” provided during the sessions was another facilitator. Echoing similar thoughts, Rose mentioned that she did not have to worry about cooking since the training provided lunch for them,“What enabled us to participate in the Happy Family training is that; they were providing lunch, so we didn’t have to worry about cooking.”

### Challenges to attendance

Some adolescents reported not having any difficulties attending the sessions. Other participants identified several factors that made it a challenge to participate in training sessions. These included household responsibilities and obligations, difficulty raising transport money despite transport refunds, long distances, and weather challenges among others. For example, Ester found it hard to attend some of the sessions due to childcare responsibilities: “*My aunt whom I live with was going somewhere and she could leave the child behind with no one so I had to stay at home.”*

Agnes missed attending a session because they had lost a relative and her mother asked her to stay at home, so she could go to the burial. Olivia mentioned that she had difficulty attending the sessions because her mother was sick. When asked if she was able to overcome the challenge, *“We weren’t able; by the time she recovered, the sessions were about to end.”*

Flora had to stay at home whenever her grandmother was away. Even after informing her grandmother of the sessions, she could not find someone else to stay home with the young children.

*Flora: Sometimes I had to stay at home with the children when my grandmother was away, so I would not attend…I would tell her that I will be going for training this date and she allows me, but she will not have someone to stay at home.*

Gloria indicated that while she was interested in attending the sessions, her parent thought that they could be spending that time working in the garden instead,* “A parent wanted to dig in the morning and evening and whenever she would think of spending time in the sessions from 9 am to 4 pm, she would consider it a waste of time.”*

Relatedly, Anisha talked about the challenge of completing the session homework for the following session due to household responsibilities and felt worried to attend the next session.

*Anisha: We would be given take-home assignments during the sessions. However, sometimes I would be so busy at home and would fail to get time to do the assignment. So, I would sometimes be anxious to come for the next session without doing the assignment.*

Several participants were challenged by weather conditions, specifically the heavy rains during the intervention period. They talked about getting to the training venue late and missing out on the beginning of the sessions. For example, Rose noted that sometimes it would rain from early morning to mid-day, making it hard for her to get to the session venue on time. Janet shared “*We found challenges with the weather changes like on days when it had rained heavily. This could make us report late for the sessions. That was the only barrier.”*

While participants were given transport refunds after each session, they had to raise the money to get to the venue. Fiona noted that in addition to the heavy rains, sometimes it was challenging to get money for her to get to the venue.

*Fiona: Sometimes it could rain heavily and also sometimes we were not able to raise transport to come for the sessions. Although we were given a refund, we found it a challenge to raise transport that would take us to school to attend the sessions.*

Difficulty raising transport money combined with long distances further prevented participants -and their family members- from either arriving to the sessions or arriving on time. For example, Violet noted that while the sessions were scheduled at the right time, they usually arrived late because they were coming from far. Joseline noted that she initially lived in a place that was farther away from where the sessions were held and the transport was costly. Hence, she missed some sessions. Eventually, she moved closer to the delivery location.

*Joseline: In the beginning I was staying in another location that was far and the transport cost was high. They used to charge me twenty five thousands [Uganda Shillings]. I missed some [sessions] because there was a time when they invited me but I was in that far location and I could not attend.*

Ruth, however, noted that she was not very interested in attending the sessions and that during the heavy rains, her guardian had to push her to attend the sessions.

*Ruth: Sometimes I liked playing more than coming to the sessions because I didn’t like them that much even though they were intended to help me. One day, it rained heavily, and the weather was bad. I didn’t want to come but then my guardian came and told me that we had to go. I went forcefully just because he had sacrificed his time to come.*

### Intervention characteristics

#### Group format

Participants mainly had positive responses to the group format. They appreciated that families could easily share their experiences, and sometimes use the group to help resolve issues. For example, Annah appreciated the group format because it facilitated knowledge sharing and learning between families.

*Annah: It was very good because you could share knowledge and be able to get different ideas about something. People would raise their hands and give different opinions about something so there was knowledge sharing and we could learn from one another.*

Ruth shared similar sentiments and added that they felt comfortable sharing with the group even when it was challenging.

*Ruth: It [the group] was amazing to us because everyone would share their family experiences and we could discuss them. Sometimes one would say that in their family, they don’t go to bed without saying prayers and we could all get to know it. We always felt comfortable sharing with our colleagues even if something was challenging.*

Mary noted that sharing experiences helped them learn from one another and made her feel comfortable. She also appreciated that everyone was given the chance to speak.

*Mary: Everyone was given a chance to share their ideas. People from different families used to share their experiences and this helped us to learn from one another on the day’s topic…The way we used to share our own experiences made me feel at ease and to make the groups lively…*

Similarly, Gloria acknowledged learning from each other and went on to discuss how being in a group helped others to gain confidence in sharing her thoughts and experiences, *“Everyone learned from another and those who weren't confident gained it in that a person could say; if this person has managed to stand and give her view, why can’t I do it also.”*

While Janet liked having other families in the group, she was less comfortable when a group member came with a family member other than the one that they usually attended with. She was worried that these people would not respect the confidentiality of the group and spread rumors in the community.

*Janet: Sitting together with other family members was not bad because we were used to one another but if someone comes with “a forged guardian” because the real guardian has not turned up, I didn’t like it. Coming with a different family member in the group was not right because after departing, they will go and start spreading rumors of what has been shared in the group which is not good.*

### Attending sessions with caregivers

Most study participants had a positive experience with attending sessions together with their caregivers. They mentioned that being with their caregivers in the sessions helped them to learn together, receive support when needed, and in some cases change their caregivers’ minds. Grace shared that they used to sit at the same desk with her caregiver, and this made it easy to consult with her caregiver in case they asked something she did not know. Janet also felt good sitting with her family members because it helped them to reconnect.

*Janet: I felt good because we take long without sitting together as family members. Everyone is usually on their own. This time helped us to come together as a family to discuss different topics and to be socially connected.*

Annet felt that attending the sessions together with her guardian helped to support the idea of starting an income generating activity. The sessions reemphasized how this was important and doable, and she was happy that her guardian was part of the training.

*Annet: These sessions did so well to train and equip us with the skills because even our guardians could not tell us that we can do this to get money. Before, they could not allow us to start up small businesses because they thought we would get spoiled. They used to tell us to wait until we finish school and start up something. After the trainings, our parents can now allow us to start up something.*

Mary thought that attending the MFG sessions with the guardian because *“there was a lot to learn for a child and a guardian, making decisions together and agreeing with one another.”*

On the other hand, while Christine enjoyed coming to the sessions with her caregiver, she did not enjoy when adolescents and caregivers completed certain activities separately. She thought that it was not the best use of anyone’s time with group members moving from one place to another.

*Christine: We could reach a session, that children would sit by themselves and parents alone, and then for me, I did not like that moment a lot because you would be seated and then they say, go and sit somewhere else, when every child as to sit with their parent and then they tell you that those that side, stay alone and even those the other side. That thing of moving and time used to be wasted there.*

### Delivery location

Sessions were conducted at schools. The research team worked closely with the school contact teachers and head teachers to identify and prepare classrooms prior to the sessions. Both Janet and Mariam noted that the location was noise-free, the classroom was spacious, and there was nothing to disrupt them from learning. Fiona shared that the venue was big enough to accommodate all the participants and that they were able to sit comfortably, **“***The venue was so good. The class was wide, and everyone had a right to sit anywhere they wanted as long as you are seated in a circle and you’re able to listen to what is being discussed.”*.

For Anisha, the venue was a central location for all and the transport costs to get there were not prohibitive.

*Anisha: The place was not bad. This is where we could all gather easily because most of us live around here and this place is in the center to where we all live so the transport cost to here was about two thousand shillings, and we could all come here.*

Racheal mentioned that the fact that sessions were conducted during the holiday break when no other students were in school, made the school a quiet place. Christine appreciated the cleanliness of the classroom as well as the light and opportunity to get fresh air: *“The class for the sessions used to be clean and we could get fresh air so well, the light was good, the class was excellent.”* However, she also pointed out that the space was small. Gloria reported that the bathrooms were far from where they conducted the sessions and was worried that somebody who might need to use the bathroom urgently would be inconvenienced.

*Gloria: The only issue was that the washrooms were very far and if someone had come having diarrhea to attend the sessions, would be inconvenienced. He/she may not even come back because the distance was too far.*

### Day and time of the sessions

Most study participants were happy with the day and times when sessions were conducted. Grace, Racheal, and Fiona noted that the session start time gave them a chance to first do some household chores before coming to the sessions. For Fiona, the day was “*favorable because we had no classes at school. The time was okay because sometimes it was in the evenings, which enabled us to first complete housework and then go for the sessions*.”

Harriet noted that the day enabled her to take a break from digging in the fields, which usually happened on Saturday. In addition, that allowed her to continue attending church on Sundays. For Mariam, the day was good because everyone was free and out of school. They did not have to attend classes, which helped to not *“disturb their brains*”. Gloria appreciated that the sessions were delivered on the weekends and during the school break. In addition, there was not much farm work at the time, which allowed caregivers to attend the sessions, *“we were on holidays and there wasn’t a lot of digging. So, parents didn’t have much work by then.”*

While some participants were happy with the day and time when the sessions were conducted, Christine felt that sometimes attending sessions on the weekend was not favorable as she was left with no time to devote to her weekend studying.

*Christine: Sometimes Saturday was not a good day for me because I used not to get time to sit and study because sometimes, I would have an examination on Monday. And on Sunday, students would be on entertainment and making noise.*

Because the sessions required both the child and the caregiver to attend at the same time, Margret felt that this was an inconvenience as they did not want to leave the household unattended.

*Margret: The time in general, was not so good because sometimes, we were only two at home. If we all leave, there is not one left behind and sometimes we go back late. But it would depend on the time you would report in the morning. It was not so good, but we endured and completed.*

Barbra thought that the start time for the sessions was too early as it made it harder for people who were coming from more distant places and would have preferred a later start time.

*Barbra: The issue was about the time. We were invited to attend the trainings in morning hours at 8:00 am. This was not favorable for people who were coming from afar. If the time was 10: 00 am at least, this would enable someone to first finish some home chores and then attend the trainings.*

### Intervention Facilitators

Participants appreciated that the facilitators were knowledgeable and prepared. They related well with the group and encouraged them to share their opinions during discussions. For example, Janet noted that the facilitators’ interactions with the group were good and they did not show any signs of favoritism. Rose talked about how facilitators encouraged everyone to share their opinions, “*They would answer any question and they were giving everybody a change to share their opinion. They would welcome every answer whether it is right or wrong.”* Similarly, Sarah shared that the facilitators were very well trained, which enabled her to share some of what she learned with other members of her family.

*Sarah: I thought of them as being well trained and conversant with the information which they had to share with us. They were able to give us very helpful information, which information we were also able to share with other members of our families.*

In addition, Mariam noted that the facilitators provided detailed explanations, they treated everyone with respect, were not rude and made sure everyone understood the content before moving on. Facilitators’ respectful attitude was mentioned by many, including Flora.

*Flora:that they were going to be very strict but they were not. I thought that they will shout at us and force us to do things, but they were peaceful. They were polite and when you talk to them, even when you give them a wrong answer, they will explain to you and make sure that you are not left behind”.*

Both Violet and Jennifer shared that the facilitators patiently repeated the content and made sure that everybody understood it. Violet added that they respected everyone’s opinion.

*Violet: They facilitated us so well. In case someone went out or had not understood, they would repeat for them such that they understand it clearly. They respected everyone’s opinion, whenever someone was speaking, they could listen carefully. They would explain every point and ensure everyone understands it. They treated us fairly without discrimination. Everyone was given chance to present their views.*

Barbra noted that having two facilitators in the session was helpful so that they could attend to different families when needed: *“The other thing that helped was having two facilitators. One would concentrate on one family and the other one would also be demonstrating something to another family as well. It was helpful to have two facilitators in a group.”*

Some participants also appreciated the facilitators’ relationships with each other. Racheal added, *"All of them worked as a team. One could advise each other, and they properly communicated with each other.”* For Ruth, the fact that they treated each other with respect and helped each other was good role-modeling for her.

### Content relevance

Participants found the content covered in both FLT &IGA and MFG sessions were relevant and helpful. For instance, Racheal shared that every time she attended a session, she learned something beneficial, "*Every time we attended, I could learn what to do. We learnt them and I didn’t take them for granted because they were beneficial*.” She went on to add that she was able to start an income generating activity that made both her and her father happy.

*Racheal: What made me happy was the fact that one was able to start an income-generating activity. My father was very happy because I didn’t have that knowledge before but when he saw that I had started trading in sugarcane, he said that his daughter had acquired great knowledge.*

Mariam appreciated the opportunity to learn about income generating activities. When asked about what in particular, she emphasized that it was important for her to understand budgeting and learn that she could start a small business even with the little money that she had.

*Mariam: I learned that even if you have little money, you can start up your small business and keep adding in slowly by slowly. I learned how to spend money. Sometimes you have to first make a budget and save and invest other money in the business. I developed more different kinds of perceptions on how to handle my business.*

Being able to have a bank account and save made Annah proud. Having a bank account at a young age made her feel different than her peers.

*Annah: I never thought that at my age I can own a bank account, that interested me so much. To save money in the bank, made me different from my friends. They see that you are different from them, at my young age yet some older people don’t even own a bank account, which makes me different.*

Sarah felt like learning to save money gave her a roadmap to solve her problems: “The skill that has been most helpful to me is saving money, which you can use to solve a problem that arises.” Violet also shared that the sessions taught her to plan for unanticipated problems.

*Violet: We learned a lot from the FLT sessions because we never knew the importance of saving for the future and unanticipated situations as well as starting up income-generating activities. They taught us that before saving, one should have the right budget. You have to first set aside money to cater for unanticipated problems and basic needs and then save the balance.*

Participants also appreciated the content they learned during the MFG sessions. For instance, Agnes shared that she learned about preventing STIs, including HIV as well as good communication and relationships among family members. Similarly, when asked about the most exciting content she learned in the sessions, Sarah shared:

*Sarah: About the different viruses that transmit sexually transmitted diseases and we all appreciated the need to protect ourselves as children. I applied the skills by avoiding behavior that puts my life at the risk of contracting sexually transmitted diseases and by appreciating the need to save money.*

Mariam was able to use the content and skills she learned during the MFG sessions to settle arguments among her siblings.

*Mariam: If there is a situation when they are quarreling, I can calm them down and tell them what we learned from the sessions. I can explain to them and sometimes they forgive each other. Sometimes when the mother is upset and she starts to quarrel, you can remind her of what was taught in the sessions.*

Anisha found the rules to be helpful. She added that her older sister who was attending the sessions changed the way she was doing certain things.

*Anisha: Since my older sister was also attending the sessions, she changed her way of doing certain things, for example, before the sessions, she would always accuse me of messing up but after the sessions, she learned that all children can mess up and that she should establish who messed up before accusing just one person.*

However, she also noted that there were instances where she chose to stay quiet in some situations instead of arguing at school and *“this made some of the students to say that I am a coward because I just kept quiet.”*

Many participants noted positive changes after the MFG sessions. Barbra shared that her parents communicated better with each other as a result of the sessions.

*Barbra: Before the trainings, mom and dad would get angry at each other and they would keep the anger for a long time but after attending the training sessions, they sit down and solve the issue every time there is a misunderstanding.*

Grace was the only participant who felt like the content was particularly helpful to her family.

*Grace: I have applied skills of having good relations at home for example gardening together, fetching water together, and in this case, we work together such that we rest at the same time. The information was good, however much it didn’t work well in our family.*

## Discussion

In this study, we explored the acceptability of the Suubi4Her combination intervention comprised of economic empowerment and family strengthening components among adolescent school-going girls in Uganda. Specifically, we explored motivations to and initial concerns about Suubi4Her program participation, barriers and facilitators to session attendance, and feedback on intervention characteristics, including content relevance, all aspects relevant to program acceptability.

Adolescent girls were motivated to participate in Suubi4Her and underlined different aspects of the program as motivating factors for them, including information about STIs and HIV as well as pregnancy. For some, HIV, STI, and pregnancy testing was also important as that would allow them to plan accordingly, as also identified by Ferrand et al. [45] as a reason for intervention acceptability among youth in Zimbabwe. Many participants presented the economic empowerment component as an initial motivation for them, specifically the matched savings. While mentioned less frequently, the potential for improving family relations was also underlined. An interesting finding was that some participants felt the program would be able to offer them the information that their caregivers would not be able to provide at home. These illustrate the initial buy-in to the program among adolescent girls and a positive anticipated affective attitude towards the program, one of the acceptability dimensions of TFA [29].

While more than half of the participants did not have any initial concerns about participating in the program, a few concerns were brought up. Some participants were worried about not being able to fulfill the requirements of the program, such as pulling together the necessary paperwork for opening bank accounts while others worried that they would not be able to save or deposit. A few participants mentioned that there was some distrust in the community or among caregivers toward the program. Community distrust has been discussed in the context of health and mental systems [46–48] as well as financial institutions [49]. This skepticism may have resulted from prior negative experiences with other service providers or programs. Alternatively, it may also stem from the scarcity of programs in the area, resulting in caregivers and community members questioning their authenticity when such programs are offered. While this misinformation did not prevent adolescent girls from participating, it is an important point to consider and adequately address prior to program implementation. Finally, the level of anticipated burden [29] expressed by adolescents was very low. Only two participants noted initial concerns about whether their caregivers would have the time to attend the program despite it being a common concern in interventions that require an extended period of time participation [50–52]. The initial enthusiasm to participate in the program and the limited hesitation on time commitment are also important indicators of initial self-efficacy, another TFA dimension, in that they felt confident they could attend the sessions.

We also inquired into the facilitators and barriers to program attendance. One of the main facilitators to attending sessions was to learn new things. Participants thought that the knowledge they gained from the sessions was too important to miss. Hence, they rearranged their other chores to make sure they could attend. Reminder calls from the study team as well as parental commitment also helped them to prioritize the sessions. These findings suggest that even when the experienced opportunity cost was high for some participants, they chose to make compromises to stay engaged. On the other side, not everybody was able to adjust. Adolescent participants mentioned family commitments and chores as a barrier to program participation, as also identified in other studies [53, 54]. Another facilitator to program attendance was the provision of incentives in the form of transport refunds and lunch/snacks. Transportation costs are widely discussed as a barrier to program participation [53, 55] as they can be prohibitive for already resource-limited families. In fact, some participants mentioned struggling with transportation costs despite the refund as they had to pay for transport upfront. Interestingly, transportation cost was still discussed as a barrier as well given that participants had to pay for transportation upfront before being reimbursed by the program. The provision of food was also raised as a factor that motivated the adolescent girls to come to the sessions, possibly pointing out its even more critical importance in low-resource settings [53]. While both these incentives have budgetary implications for program implementation, they should be considered for interventions in limited-resource settings. The findings on the barriers and facilitators to participation also illustrate the variation in the experienced burden across the participants.

Adolescent girls provided feedback on the different characteristics of the program. Overall, they found the location and time of session delivery convenient. They appreciated the group format as that allowed them to learn from other families. They also welcomed attending the sessions with their caregivers as that provided the opportunity to spend time and learn together. Intervention facilitators were perceived as helpful, inclusive, and respectful. Finally, adolescent girls found the session contents highly relevant to their families’ needs and noted positive changes in their families, including saving and improved family relations. Perceived relevance of content has been cited as an indicator of intervention acceptability in other studies as well [56–58]. While ethicality [29] did not emerge as a theme in our data, it is likely that participants would have stopped attending if the program was in direct conflict with their value system. On the other hand, participants’ discussion of the content and skills they learned as a result of the sessions point to intervention coherence. While some participants noted that the skills did not always work out perfectly in real-life situations, many noted that they were able to use them in their lives, showing participants’ self-efficacy in performing the skills [29]. Increased knowledge and positive change have been cited as a reason given for acceptability in several studies conducted among adolescents in SSA, including in Uganda [56, 59–62]. Additionally, the improvements noted by the participants speak directly to the perceived effectiveness of the intervention, another TFA dimension. Given that participants expected to improve knowledge on health, saving/budgeting as well as family relations at the time of enrolment and noted changes in all these areas upon intervention completion, one can conclude that there was an overlap between anticipated and experienced perceived effectiveness.

Overall, our findings suggest that adolescent girls found the Suubi4Her combination intervention highly acceptable, an important and promising step for its uptake and scalability in the region and beyond, while also pointing to certain barriers that need to be taken into account. Study results need to be interpreted in light of study limitations. The qualitative interviews were conducted cross-sectionally upon completion of the intervention. Hence, the data do not capture any changes that may occur in adolescents’ perspectives on their experiences with the intervention over time. Additionally, the TFA was used at the stage of data analysis, rather than at the stage of developing the interview protocol. Relatedly, certain constructs such as ethicality and intervention coherence were not explored in-depth during data collection. However, these findings contribute to the scarce literature on intervention acceptability among adolescents in sub-Saharan Africa. Another strength of the study is the sampling strategy that allowed us to capture the experiences of both those who fared well on the targeted intervention outcomes as well as those who did not.

## Conclusion

Interventions targeting adolescent health and social outcomes are more likely to succeed if they are found acceptable by the youth that they are intended for. Our findings provide important insights into the acceptability of a combination intervention among adolescent girls, and relatedly, potential for sustainability and scalability. These findings also have important programmatic and policy implications in Uganda, especially given the higher rates of HIV prevalence among adolescent girls in the country. The Uganda Government and Ministry of Health identify adolescent girls and young women as one of the priority groups targeted by combination HIV prevention, care and treatment, and social support interventions, as outlined in strategic action 3.2.2 of the National Strategic Plan for HIV and AIDS [63]. As such, it is important to consider incorporating evidence-based interventions with high acceptability into national HIV prevention programs and policies. The study results are particularly relevant as they are guided by a theoretical framework for acceptability, explore the acceptability of an intervention tested as part of a randomized clinical trial, and contribute to the scarce literature on intervention acceptability research with adolescents in LMICs.

## Data Availability

Given that the qualitative data contains potentially identifying and sensitive information, the data will not be made publicly available as it would violate the privacy and confidentiality of the study participants. The excerpts of the transcripts relevant to the study have been included in the manuscript. Deidentified data will be available upon request from the first author or the Institutional Review Board at Washington University in St. Louis at HRPO@wustl.edu.

## Abbreviations

FLT: Financial literacy training
IGAs: Income-generating activities
LMICs: Low and middle-income countries
MFG: Multiple family group
NIMH: National Institute of Mental Health
PrEP: Pre-exposure prophylaxis
SSA: Sub-Saharan Africa
STI: Sexually transmitted infection
TFA: Theoretical framework of acceptability
YDA: Youth development account

## Code availability

NA

## References

[1] UNAIDS (2021). Confronting Inequalities: Lessons for pandemic responses from 40 years of AIDS. Retrieved from https://www.unaids.org/sites/default/files/media_asset/2021-global-aids-update_en.pdf

[2] Cho H, Hallfors DD, Mbai II, Itindi J, Milimo BW, Halpern CT, Iritani BJ (2011). Keeping Adolescent Orphans in School to Prevent Human Immunodeficiency Virus Infection: Evidence From a Randomized Controlled Trial in Kenya. J Adolesc Health.

[3] Glynn, J. R., Caraël, M., Auvert, B., Kahindo, M., Chege, J., Musonda, R., Kaona, F., Buvé, A., & Study Group on the Heterogeneity of HIV Epidemics in African Cities (2001). Why do young women have a much higher prevalence of HIV than young men? A study in Kisumu, Kenya and Ndola. Zambia AIDS.

[4] Gregson S, Garnett GP (2000). Contrasting gender differentials in HIV-1 prevalence and associated mortality increase in eastern and southern Africa: Artefact of data or natural course of epidemics?. AIDS.

[5] Hallfors D, Cho H, Rusakaniko S, Iritani B, Mapfumo J, Halpern C (2011). Supporting adolescent orphan girls to stay in school as HIV risk prevention: Evidence from a randomized controlled trial in Zimbabwe. Am J Public Health.

[6] Nobelius A-M, Kalina B, Pool R, Whitworth J, Chesters J, Power R (2011). Sexual partner types and related sexual health risk among out-of-school adolescents in rural south-west Uganda. AIDS Care.

[7] Pettifor AE, Levandowski BA, MacPhail C, Padian NS, Cohen MS, Rees HV (2008). Keep them in school: The importance of education as a protective factor against HIV infection among young South African women. Int J Epidemiol.

[8] Gunn JKL, Roth AM, Center KE, Wiehe SE (2016). The Unanticipated Benefits of Behavioral Assessments and Interviews on Anxiety, Self-Esteem and Depression Among Women Engaging in Transactional Sex. Community Ment Health J.

[9] Meier A, Erickson GA, McLaughlin H (2016). Older Sexual Partners and Adolescent Females’ Mental Health. Perspect Sex Reprod Health.

[10] Barhafumwa B, Dietrich J, Closson K, Samji H, Cescon A, Nkala, B.,…& the Botsha Bophelo Adolescent,  (2016). High prevalence of depression symptomology among adolescents in Soweto, South Africa associated with being female and cofactors relating to HIV transmission. Vulnerable Children and Youth Studies.

[11] Agbor J. (2012) Poverty, inequality and Africa's education crisis. Brookings, DC. Retrieved from: https://www.brookings.edu/opinions/poverty-inequality-and-africas-education-crisis/.

[12] The World Bank (2009). Abolishing school fees in Africa: Lessons from Ethiopia, Kenya, Malawi and Mozambique. Retrieved from: http://documents.worldbank.org/curated/en/780521468250868445/Abolishing-school-fees-in-Africa-lessons-from-Ethiopia-Ghana-Kenya-Malawi-and-Mozambique.

[13] Colclough C, Rose P, Tembon M (2000). Gender inequalities in primary schooling: the roles of poverty and adverse cultural practice. Int J Educ Dev.

[14] Ombati V, Ombati M (2012). Gender inequality in education in sub-Saharan Africa. Journal of Women's Entrepreneurship and Education.

[15] Ssewamala FM, Wang JSH, Karimli L, Nabunya P (2011). Strengthening universal primary education in Uganda: the potential role of an asset-based development policy. Int J Educ Dev.

[16] Ssewamala FM, Ismayilova L, McKay M, Sperber E, Bannon W, Alicea S (2010). Gender and the effects of an economic empowerment program on attitudes toward sexual risk-taking among AIDS-orphaned adolescent youth in Uganda. J Adolesc Health.

[17] UNESCO. New global education goals must prioritize girls. Education for all global monitoring report [Internet]. UNESCO; 2014. Available from: http://www.unesco.org/new/en/media-services/single%20view/news/new_global_education_goals_must_prioritize_girls/#.Vnl3dvkrJph.

[18] Jukes M, Simmons S, Bundy D (2008). Education and vulnerability: the role of schools in protecting young women and girls from HIV in southern Africa. AIDS.

[19] Hill HD, Morris P, Gennetian LA, Wolf S, Tubbs C (2013). The consequences of income instability for children's well-being. Child Development Perspectives.

[20] Nabunya P, Ssewamala FM, Ilic V (2014). Family economic strengthening and parenting stress among caregivers of AIDS-orphaned children: results from a cluster randomized clinical trial in Uganda. Child & Youth Services Review.

[21] Wang JSH, Ssewamala FM, Han CK (2014). Family economic strengthening and mental health functioning of caregivers for AIDS-affected children in rural Uganda. Vulnerable children and youth studies.

[22] McNeely C, Shew ML, Beuhring T, Sieving R, Miller BC, Blum RW (2002). Mothers’ influence on the timing of first sex among 14-and 15-year-olds. J Adolesc Health.

[23] Resnick MD, Bearman PS, Blum RW, Bauman KE, Harris KM, Jones J, Tabor J, Beuhring T (1997). Protecting adolescents from harm: findings from the national longitudinal study on adolescent health. JAMA.

[24] Amerikaner M, Monks G, Wolfe P, Thomas S (1994). Family interaction and individual psychological health. J Couns Dev.

[25] Hurst JL, Widman L, Maheux, AJ, Evans-Paulson R, Brasileiro J, Lipsey N. Parent–child communication and adolescent sexual decision making: An application of family communication patterns theory. J of Fam Psychol. 2022;36(3):449–57. 10.1037/fam0000916. 10.1037/fam0000916 34472938

[26] Kotchick BA, Dorsey S, Miller KS, Forehand R (1999). Adolescent sexual risk-taking behavior in single-parent ethnic minority families. J Fam Psychol.

[27] Miller KS, Forehand R, Kotchick BA (1999). Adolescent sexual behavior in two ethnic minority samples: the role of family variables. J Marriage Fam.

[28] Somefun, O. D., Casale, M., Haupt Ronnie, G., Desmond, C., Cluver, L., & Sherr, L. (2021). Decade of research into the acceptability of interventions aimed at improving adolescent and youth health and social outcomes in Africa: A systematic review and evidence map. BMJ Open, 11(12), e055160. 10.1136/bmjopen-2021-055160 10.1136/bmjopen-2021-055160 PMC8689197 34930743

[29] Sekhon, M., Cartwright, M., & Francis, J. J. (2017). Acceptability of healthcare interventions: An overview of reviews and development of a theoretical framework. BMC Health Services Research, 17(1). 10.1186/s12913-017-2031-8 10.1186/s12913-017-2031-8 PMC5267473 28126032

[30] Yeager DS, Dahl RE, Dweck CS (2018). Why Interventions to Influence Adolescent Behavior Often Fail but Could Succeed. Perspect Psychol Sci.

[31] Baron D, Scorgie F, Ramskin L, Khoza N, Schutzman J, Stangl A, Harvey S, Delany-Moretlwe S; EMPOWER study team. "You talk about problems until you feel free": South African adolescent girls' and young women's narratives on the value of HIV prevention peer support clubs. BMC Public Health. 2020 Jun 26;20(1):1016. doi: 10.1186/s12889-020-09115-4. PMID: 32590969; PMCID: PMC7320560. 10.1186/s12889-020-09115-4 PMC7320560 32590969

[32] Jackson-Gibson, M., Ezema, A. U., Orero, W., Were, I., Ohiomoba, R. O., Mbullo, P. O., & Hirschhorn, L. R. (2021). Facilitators and barriers to HIV pre-exposure prophylaxis (PrEP) uptake through a community-based intervention strategy among adolescent girls and young women in Seme Sub-County, Kisumu, Kenya. BMC Public Health, 21(1). 10.1186/s12889-021-11335-1 10.1186/s12889-021-11335-1 PMC8252310 34210288

[33] Muhumuza R, Ssemata AS, Kakande A, Ahmed N, Atujuna M, Nomvuyo M, Bekker L-G, Dietrich JJ, Tshabalala G, Hornschuh S, Maluadzi M, Chibanda-Stranix L, Nematadzira T, Weiss HA, Nash S, Fox J, Seeley J (2021). Exploring Perceived Barriers and Facilitators of PrEP Uptake among Young People in Uganda, Zimbabwe, and South Africa. Arch Sex Behav.

[34] Byansi W, Ssewamala FM, Neilands TB, Sensoy Bahar O, Nabunya P, Namuwonge F, McKay MM (2022). The Short-Term Impact of a Combination Intervention on Depressive Symptoms Among School-Going Adolescent Girls in Southwestern Uganda: The Suubi4Her Cluster Randomized Trial. J Adolesc Health.

[35] Nabunya P, Byansi W, Muwanga J, Bahar OS, Namuwonge F, Ssentumbwe V, Ssewamala FM (2022). Family Factors and Gender Norms as Protective Factors Against Sexual Risk-Taking Behaviors Among Adolescent Girls in Southern Uganda. Global Social Welfare.

[36] Uganda Aids Commission Secretariat. Factsheet- Facts on HIV and AIDS in Uganda 2021 (Based on Data ending 31st december 2020). 2021; https://uac.go.ug/media/attachments/2021/09/13/final-2021-hiv-aids-factsheet.pdf.

[37] Ssewamala, F. M., Bermudez, L. G., Neilands, T. B., Mellins, C. A., McKay, M. M., Garfinkel, I., Sensoy Bahar, O., Nakigozi, G., Mukasa, M., Stark, L., Damulira, C., Nattabi, J., & Kivumbi, A. (2018). Suubi4Her: A study protocol to examine the impact and cost associated with a combination intervention to prevent HIV risk behavior and improve mental health functioning among adolescent girls in Uganda. BMC Public Health, 18(1). 10.1186/s12889-018-5604-5 10.1186/s12889-018-5604-5 PMC5989412 29871619

[38] McKay MM, Gonzales JJ, Stone S, Ryland D, Kohner K (1995). Multiple family therapy groups: a responsive intervention model for inner city families. Social Work with Groups.

[39] Sensoy Bahar O, Byansi W, Kivumbi A, Namatovu P, Kiyingi J, Ssewamala FM, McKay MM, Nyoni T (2020). From, “4Rs and 2Ss” to “Amaka Amasanyufu”(Happy Families): Adapting a US-based Evidence-Based Intervention to the Uganda Context. Fam Process.

[40] Beck AT, Steer RA, Brown GK (1987). Beck depression inventory.

[41] Strauss A, Corbin J (1998). Basics of qualitative research techniques.

[42] Corbin J, Strauss A (2014). Basics of qualitative research: Techniques and procedures for developing grounded theory.

[43] Lincoln YS, Guba EG (1985). Naturalistic inquiry.

[44] Padgett DK (2008). Qualitative methods in social work research (Vol. 36).

[45] Ferrand RA, Trigg C, Bandason T (2011). Perception of risk of vertically acquired HIV infection and acceptability of providerinitiated testing and counseling among adolescents in Zimbabwe. Am J Public Health.

[46] Contractor LF, Celedonia KL, Cruz M, Douaihy A, Kogan JN, Marin R, Stein BD (2012). Mental health services for children of substance abusing parents: voices from the community. Community Ment Health J.

[47] Field S, Honikman S, Abrahams Z (2020). Adolescent mothers: a qualitative study on barriers and facilitators to mental health in a low-resource setting in Cape Town, South Africa. African Journal of Primary Health Care and Family Medicine.

[48] Hodge LM, Turner KM (2016). Sustained implementation of evidence-based programs in disadvantaged communities: A conceptual framework of supporting factors. Am J Community Psychol.

[49] Baidoo ST, Akoto L (2019). Does trust in financial institutions drive formal saving? Empirical evidence from Ghana. Int Soc Sci J.

[50] Geng EH, Nash D, Kambugu A, Zhang Y, Braitstein P, Christopoulos KA, Muyindike W, Bwana MB, Yiannoutsos CT, Petersen ML, Martin JM (2010). Retention in care among HIV-infected patients in resource-limited settings: Emerging insights and new directions. Curr HIV/AIDS Rep.

[51] Shenderovich Y, Eisner M, Cluver L, Doubt J, Berezin M, Majokweni S, Murray AL (2018). What affects attendance and engagement in a parenting program in South Africa?. Prev Sci.

[52] Yeasmin, F., Winch, P. J., Hwang, S. T., Leontsini, E., Jahir, T., Das, J. B., ... & Rahman, M. (2021). Exploration of attendance, active participation, and behavior change in a group-based responsive stimulation, maternal and child health, and nutrition intervention. The American Journal of Tropical Medicine and Hygiene, 104(4), 1586. doi: 10.4269/ajtmh.20-0991 10.4269/ajtmh.20-0991 PMC8045643 33534769

[53] Mathews C, Duby Z, Bunce B, van Blydenstein N, Bergh K, Ambrose A, Jonas K (2022). Safe spaces for beneficiaries of a combination HIV prevention intervention for adolescent girls and young women in South Africa: access, feasibility, and acceptability. BMC public health..

[54] McClinton Appollis T, Duby Z, Jonas K, Dietrich J, Maruping K, Abdullah F, Mathews C (2021). Factors influencing adolescent girls and young women’s participation in a combination HIV prevention intervention in South Africa. BMC public health..

[55] Murray, L. K., Skavenski, S., Michalopoulos, L. M., Bolton, P. A., Bass, J. K., Familiar, I., ... & Cohen, J. (2014). Counselor and client perspectives of trauma-focused cognitive behavioral therapy for children in Zambia: a qualitative study. Journal of Clinical Child & Adolescent Psychology, 43(6), 902–914. doi: 10.1080/15374416.2013.859079 10.1080/15374416.2013.859079 PMC4087094 24400677

[56] Banda E, Svanemyr J, Sandøy IF, Goicolea I, Zulu JM (2019). Acceptability of an economic support component to reduce early pregnancy and school dropout in Zambia: a qualitative case study. Glob Health Action.

[57] Patel V, Chowdhary N, Rahman A, Verdeli H (2011). Improving access to psychological treatments: lessons from developing countries. Behav Res Ther.

[58] Parker, L., Maman, S., Pettifor, A., Chalachala, J. L., Edmonds, A., Golin, C. E., ... & Behets, F. (2013). Feasibility analysis of an evidence-based positive prevention intervention for youth living with HIV/AIDS in Kinshasa, Democratic Republic of the Congo. AIDS education and prevention: official publication of the International Society for AIDS Education, 25(2), 135. doi: 10.1521/aeap.2013.25.2.135 10.1521/aeap.2013.25.2.135 PMC3777231 23514081

[59] Chirwa-Kambole E, Svanemyr J, Sandøy I, et al. Acceptability of youth clubs focusing on comprehensive sexual and reproductive health education in rural Zambian schools: a case of central Province. BMC Health Serv Res. 2020;20:42. 10.1186/s12913-020-4889-0. 10.1186/s12913-020-4889-0 PMC6966797 31948452

[60] Kansiime C, Hytti L, Nalugya R (2020). Menstrual health intervention and school attendance in Uganda (MENISCUS-2): a pilot intervention study. BMJ Open.

[61] Sabben G, Mudhune V, Ondeng'e K (2019). A smartphone game to prevent HIV among young Africans (Tumaini): assessing intervention and study acceptability among adolescents and their parents in a randomized controlled trial. JMIR Mhealth Uhealth.

[62] Ybarra ML, Bull SS, Prescott TL (2014). Acceptability and feasibility of CyberSenga: an Internet-based HIV-prevention program for adolescents in Mbarara. Uganda AIDS Care.

[63] National HIV and AIDS Strategic Plan 2020/2021–2024/2025: Ending the HIV and AIDS epidemic: Communities at the forefront: Kampala: Uganda AIDS Commission; 2020. Retrieved from https://uac.go.ug/index.php?option=com_content&view=article&id=34:hiv-prevention-11&catid=8&Itemid=101

